# Genetic analyses of the structural protein E2 bovine viral diarrhea virus isolated from dairy cattle in Yogyakarta, Indonesia

**DOI:** 10.14202/vetworld.2024.1562-1574

**Published:** 2024-07-21

**Authors:** S. U. Khan, Hastari Wuryastuty, M. H. Wibowo, Sarmin Sarmin, S. H. Irianingsih

**Affiliations:** 1 Doctoral Study Program, Faculty of Veterinary Medicine, Universitas Gadjah Mada, Yogyakarta, Indonesia; 2 Department of Veterinary Internal Medicine, Faculty of Veterinary Medicine, Universitas Gadjah Mada, Yogyakarta, Indonesia; 3 Department of Veterinary Microbiology, Faculty of Veterinary Medicine, Universitas Gadjah Mada, Yogyakarta, Indonesia; 4 Department of Veterinary Physiology, Faculty of Veterinary Medicine, Universitas Gadjah Mada, Yogyakarta, Indonesia; 5 Disease Investigation Center Wates, Yogyakarta, Indonesia

**Keywords:** bovine viral diarrhea virus, cysteine mutation, E2 protein, serine, V11 bovine viral diarrhea virus1, V16 bovine viral diarrhea virus1

## Abstract

**Background and Aim:**

Bovine viral diarrhea (BVD), a highly pathogenic ribonucleic acid (RNA) virus, causes devastating financial losses and reproductive deaths among dairy cattle in Yogyakarta and globally. This study aimed to identify point mutations within the E2 structural protein of the acquired BVD virus (BVDV) isolates using genetic analysis.

**Materials and Methods:**

The study period shows that we performed the research in 2023. We collected 118 serum samples from 2019 to 2023, among which only 10 BVDV positive were used and 108 were negative lacking the BVDV antigen. An anti-Erns monoclonal antibody-coated protein was used in indirect antigen capture enzyme-linked immunosorbent assay (I-ACE) to detect the BVD antigen present in positive BVDV serum specimens. In the initial step of the two-step reverse transcription polymerase chain reaction, the enzyme (superscript III reverse transcriptase) and the primer (random hexamer) were used to convert the RNA of the BVDV into complementary deoxyribonucleic acid (cDNA) during the process of reverse transcription. The final step involved the amplification of the E2 gene of the resultant BVDV cDNA through gene-specific primers (E2_fwd: 5′-TGGTGGCCTTATGAGAC-3′ and P7_rev: 5′-CCCATCATCACTATTTCACC-3′) and enzyme (platinum taq DNA polymerase high fidelity). For conducting Sanger sequencing, those 3 BVDV-1-positive isolates (about 2.6% of all isolates) were selected as a typical specimen for each site and year between 2019 and 2023 using a proportional computation. Therefore, only two BVDV isolates with complete genomes were chosen to perform their homological and genetic analysis based on the E2 gene by means of Blast and MEGA Version 11 in addition to the Bioedit 7.2.5 program.

**Results:**

By applying phylogenetic analysis relying on the E2 gene, a sum of 1011 nucleotides of the BVDV-1 isolates derived from each of the two BVDV-1 Indonesian isolates (n = 2) and its 23 reference BVDV strains were acquired from the National Center for Biotechnology Information (NCBI) database. The findings of the genetic analysis inside the phylogenetic tree revealed that the two BVDV Indonesian isolates were clustered into BVDV-1a subgenotype, while the reference BVDV strains were clustered into the five BVDV subgenotype, BVDV-1a (n = 6), BVDV-1b (n = 3), BVDV-1c (n = 11), BVDV-1m (n = 1), and BVDV-1n (n = 2). The branch exists in phylogenetic tree located before the division of our two BVDV isolates was divided into two branches with the same maximum bootstrap values of 99%, indicating a high degree of confidence, was seen. Next, we observed the branch near our study samples, which displayed the bootstrap value of 100, indicating that our 02 isolates were identical. In both isolates, V11 BVDV1/Indonesia/Yogyakarta/2023 and V16 BVDV1/Indonesia/Yogyakarta/2023 with GenBank accession numbers PP836388 and PP836389, respectively, conserved D7E residues were mutated as well as cysteine changed/altered into serine (S) was identified at amino acid position 201.

**Conclusion:**

We identified two isolates of BVDV belonging to the BVDV-1a subgenotype. Our findings indicate that the conserved D7E residues of isolates V11 BVDV1/Indonesia/Yogyakarta/2023 and V16 BVDV1/Indonesia/Yogyakarta/2023 were altered. The Indonesian BVDV isolates exhibited a cysteine to serine mutation at amino acid position 201, leads to vaccination failure, range of animal’s host will increase, and diagnostic kit will not be effective.

## Introduction

The bovine viral diarrhea virus (BVDV) is responsible for bovine viral diarrhea (BVD) within the *Flaviviridae* family. The BVDV belongs to the four accepted species within the *pestivirus* genus [1]. This disease is distinguished by an increased prevalence of other health conditions [2]. In 1946, this disease was endemic and reported for the 1^st^ time in cattle (*Bos indicus* and *Bos taurus*) in North America [3, 4], and it was described 18 years later in Switzerland [5]. In South Sulawesi, Indonesia, the first case of BVD was discovered in 1989, impacting milking cows in the Bali region [6]. In October 1989, reports of further incidents occurred in West Kalimantan following the importation of Bali cattle from South Sulawesi [6]. The continents where BVDV is currently found include Africa, Australia, America, Europe, and Asia, including Indonesia [6, 7]. The prevalence had risen to 46% by 2020 [8]. The genus *pestivirus* belongs to the *Flaviviridae* family of viruses, which also includes the pronghorn antelope virus, rare Bungowannah virus, border disease virus, classical swine fever virus (CSFV), Aydin-like pestiviruses, HoBi-like pestiviruses, and atypical porcine *pestivirus* [9, 10]. A *pestivirus*, which is a positive-sense single-stranded ribonucleic acid (RNA) virus from the *Flaviviridae* family’s *pestivirus* genus, is responsible for diarrhea in various domestic and wild animals. The four species of *pestivirus* are the viruses that cause classical swine fever (*pestivirus* C), border disease (*pestivirus* D), bovine viral diarrhea due to Type 1 BVDV virus (*pestivirus* A, BVDV-1), and BVDV Type 2 (*pestivirus* B, BVDV-2) [11]. New *pestivirus* genus members have been identified by Rivas *et al*. [11]. Through nucleotide sequence analysis, the distinct subtypes of BVDV-1 (BVDV-1a–BVDV-1u) and BVDV-2 (BVDV-2a–BVDV-2d) have been identified [12]. The BVDV can be separated into two biotypes, non-cytopathic (NCP) and cytopathic (CP), and three genotypes, BVDV-1, BVDV-2, and BVDV-3 and the pathophysiology and epidemiology of BVDV are influenced by the CP and NCP biotypes observed in both BVDV-1 and BVDV-2 genotypes [13]. By inhibiting the intrinsic immune reaction and making the virus more accessible to live on in cattle for the whole of their lives, NCP-BVDV might cause persistent infection (PI) in these animals [14]. Bovine viral diarrhea is a prevalent global illness resulting from infection by either BVDV-1 or BVDV-2 [15]. The primary impacts of BVDV infection inside the body of the cattle are immunosuppression and pathological issues with respiratory and reproductive health [15]. Clinical signs of BVD encompass hemorrhagic syndrome, diarrhea, respiratory disorders, infertility, congenital anomalies, and mucosal diseases [16]. The three main pestiviruses that infect bovine animals are *pestivirus* bovis (BVDV1), *pestivirus* tauri (BVDV2), and *pestivirus* H (also known as HoBi-like *pestivirus* or BVDV3) [16–23]. Four subtypes of 2a–2d are present in BVDV-2, whereas BVDV-1 includes a minimum of 22 subgenotype of 1a–1v [24–27]. The BVDV is categorized into 19 distinct species of pestiviruses by the International Committee on Taxonomy of Viruses (ICTV). The BVDV genome, with a size of about 12.3 kb, comprises a solitary open reading frame (ORF) along with the short 5’ and 3’ untranslated regions that surround it [28, 29]. In addition, BVDV-1 (1a–1x) has at least 24 subgenotypes, and BVDV-2 (2a–2e) has five subgenotype [30–33]. Almost 40 artiodactyl species other than cattle are infected by BVDV. These include sheep, camelids, antelopes, and deer, which can also be infected and produce similar clinical symptoms to cattle [34, 35]. The BVDV substantially impacts the financial condition of infected livestock. Animals infected with BVDV may experience fertility issues, decreased milk production, stunted growth, chronic infection, weakness, and lethargy [34]. The virus negatively impacts the immune system of cattle and ruminants, leading to financial losses due to immunological dysfunction. Lymphoid decline, which ranges in severity depending on the virulence of BVDV strains, contributes to the immunological dysfunction linked to BVDV [35, 36]. BVDV impairs alveolar macrophages’ function by hindering complement Fc receptors’ proliferation and chemokine generation [36]. BVDV alters immunological mediators and cell function, thereby accelerating the rate of viral disease [9]. Neutrophils become less microbicidal during BVDV infection [9]. Translation of a single polyprotein can start through the internal ribosome entry site (IRES) found in the 5′-UTR of BVDV. Two extremely diverse areas are found in each of the three helices that constitute the IRES [37]. The 3′-UTR has sites for binding to many host cell microRNAs and, in place of the poly-A tail, conserved stem-loops [37]. Eight non-structural and four structural proteins are produced through post-translational processing of one large polyprotein encoded by the ORF. Eight non-structural proteins (Npro, P7, NS2, NS3, NS4A, NS4B, NS5A, and NS5B) and four structural proteins (C, Erns, E1, and E2) are produced by the BVDV-encoding polyprotein. The E2 protein of BVDV, with significant immune-activating capabilities, plays a crucial role in vaccine production. Effective BVD management relies significantly on biosecurity measures, including herd separation of PI animals and vaccination within live tissues of sensitive livestock. Effective identification of PI animals in BVDV control initiatives is accomplished through the enzyme-linked immunosorbent assay (ELISA) antigen capture method using either Erns monoclonal antibodies or NS3 antigens for tissue and serum antigen detection [38, 39].

E2 is one of the structural proteins of BVDV expressed on the surface of the virion, with a molecular mass of 55 kDa and composed of 373 amino acids. The main immunogenic protein of the virus, E2, is found in the BVDV envelope. Host cellular receptors, such as CD-46 and low-density lipoprotein receptor (LDL-R), have made easier for the virus to adhere to them by the E2 glycoprotein [40]. For BVDV, two distinct cellular receptor molecules namely, CD46 and LDL-R have been identified. A basic necessary binding platform made up of two short peptides on antiparallel beta strands in complement control protein 1 allowed the virus to connect to CD46. A 100-fold increase in BVDV susceptibility was associated with the expression of CD46 in pig cells. The inhibiting impact of an anti-LDL receptor antibody on BVDV infection of bovine turbinate (BT) cells showed a significant function for the LDL receptor in BVDV entrance. Furthermore, it was demonstrated that a bovine cell line devoid of any functional LDL receptor is totally immune to BVDV infection [40]. The main target of neutralizing antibodies of the host is the E2 glycoprotein. The development of a subunit vaccine against BVDV could be possible with the use of E2 as an outstanding antigen candidate [40]. Regular vaccination is essential for controlling and eliminating BVD in areas with high occurrence of the disease. The most recent epidemiological studies [40–44] suggest that BVDV-1 predominates in Asia.

Our research focused on serum samples recovered from dairy cattle, and the district of Yogyakarta was used as a location. The main structural constituent of the BVDV virion is the E2 glycoprotein, which is responsible for promoting the binding of the virus with specialized receptors localized on the cell surface of different animal species, ultimately eliciting immune responses against viral infection inside animal hosts. The detailed assortment of E2 coding sequences of BVDV-recovered genotypes will be illuminated, which will offer data for BVD control and the development of subunit vaccine design in the future in Yogyakarta, Indonesia. The study aimed to genetically assess the collected BVDV isolates from Indonesian dairy cows between 2019 and 2023 based on their E2 protein structure. The genetic analysis relied on identifying a specific point mutation in the E2 structural protein of the BVDV isolates. The study’s hypothesis is that E2 structural protein’s genetic sequence from the BVD virus in Yogyakarta, Indonesia, shows no distinct mutations that could affect the virus’s virulence, chance to enter a new animal’s host, effectiveness of the vaccines, screening potential of the diagnostic tests, and transmissibility in the populations of dairy cattle in the Yogyakarta, Indonesia.

## Materials and Methods

### Ethical approval

Ethical clearance was not necessary since this study utilized only serum samples obtained from diagnostic laboratories and did not involve human or animal subjects.

### Study period and location

This study was conducted from July to December 2023. The processing of the 10 positive serum samples of BVDV, commencing from their RNA extraction until amplification of the specific BVDV E2 gene was carried out at the Laboratory of Biochemistry, Department of Biotechnology, Gadjah Mada University, Yogyakarta – Indonesia.

### Serum collection

For this study, we have a total of 118 serum samples among which 10 serum samples that tested positive for BVDV antigen, while the remaining 108 were BVDV antigen negative, and were acquired from Laboratorium Ilmu Penyakit Dalam, Klinik Hewan Kuningan Fakultas Kedokteran Hewan, Yogyakarta, Indonesia. Sera of sufficient quality and high concentration, tested positive for the BVDV antigen using I-ACE, were stored at –80^O^C until evaluation. The test identified infected dairy cattle carrying the BVDV antigen.

### Serological examination

I-ACE test for BVDV was conducted using BVDV antigen test kit/serum plus (IDEXX Laboratories - USA) to distinguish between infected and uninfected dairy cows. An anti-Erns monoclonal antibody was used to recognize the BVD antigen present in BVDV infected animals. The number of optical density (OD) in the serological examination determined the livestock infected with BVDV. The corrected OD value of ≥0.3 in positive BVDV serum samples and ≤0.3 in negative BVDV isolates indicates the presence/absence of BVDV antigen based on the standard parameter (Table-1).

**Table-1:** Screening of serum through I-ACE with OD value.

S. No.	Name of BVDV positive isolates	Targeted animal’s species	Type of the sample collected	Screening of serum through I-ACE			

				BVDV positive isolates	OD Value	BVDV negative isolates	OD value
1	BVDV 23–11	Dairy cattle	Serum	10	≥0.3	108	≤0.3
2	BVDV 23–12						
3	BVDV 23–13						
4	BVDV 23–14						
5	BVDV 23–15						
6	BVDV 23–16						
7	BVDV 23–17						
8	BVDV 23–18						
9	BVDV 23–19						
10	BVDV 5060–2						

ELISA=Enzyme-linked immunosorbent assay, I-ACE=Indirect antigen capture enzyme-linked immunosorbent assay, BVDV=Bovine viral diarrhea virus, OD=Optical density

### RNA extraction

The previously 10 BVDV-positive serum samples (BVDV 23–11, BVDV 23–12, BVDV 23–13, BVDV 23–14, BVDV 23–15, BVDV 23–16, BVDV 23–17, BVDV 23–18, BVDV 23–19, and BVDV–5060-2) recovered from dairy cattle in Yogyakarta, Indonesia and screened through I-ACE (Table-1). For convenience, we gave primary names to the above-mentioned BVDV serum specimens before Sanger’s sequencing, and we assigned different names to our BVDV isolates during phylogenetic analysis. These positive serum specimens will be used to isolate BVDV RNA using a QIAamp viral RNA kit (Qiagen, Qiagen Biotech Ltd. - Germany) as recommended by the manufacturer. The temperature (−80°C) at which the RNA product was kept until it was used for the synthesis of complementary deoxyribonucleic acid (cDNA).

### Two-step reverse transcription-polymerase chain reaction (two-step RT-PCR)

The production of cDNA from RNA was performed in the first step of RT-PCR using Superscript III Reverse Transcriptase (Invitrogen™, USA) along with the use of the random hexamer primer (Invitrogen™) at an optimized temperature of 47°C. The second step of RT-PCR involved the use of Platinium Taq DNA polymerase high fidelity (Invitrogen - Thermo Fisher Scientific Inc., Waltham, Massachusetts, USA) and gene-specific primers (E2_fwd: 5′-TGGTGGCCTTATGAGAC-3′ and P7_rev: 5′-CCCATCATCACTATTTCACC-3′) (Table-2) to amplify the E2 gene of the resulting cDNA [45]. The protocol previously applied by Lang *et al*. [45] involves the thermal cycler machine being adjusted before use with initial denaturation (94°C–2 min and 30 s), followed by 25–35 cycles of 94°C for 30 s during final denaturation, 30 s of annealing at 55°C, 1 min and 30 s of extension at 68°C, and indefinitely time of hold at 4°C (Table-3) [45].

**Table-2:** Series of pair of primers for cycling of E2 open reading frame.

Primer	Sequence 5′–3′	Position	BVDV gene E2 (at nucleotide level) length obtained through MEGA-11	Position of coding’s region of E2 gene	Size of E2 gene (at amino acid level) length obtained through MEGA-11	Target BVDV gene size after gel electro- phoresis	Reference
E2_fwd P7_rev	TGGTGGCCTTATGAGAC CCCATCATCA CTATTTCACC	2345– 3356	1011 bp	2881– 3218	337 bp	E2 gene 1464 bp	[45]

BVDV=Bovine viral diarrhea virus

**Table-3:** The 2^nd^ step in the two-step RT-PCR’s second phase, which involves amplifying the E2 gene, kept at different temperatures for varying amounts of time [45].

S. No.	Amplification of the E2 gene through two-step-RT PCR	Temperature	Time duration
1	Initial denaturation	94	2 min–30 s
2	Final denaturation	94	30 s
3	Annealing	53	30 s
4	Extension	68	90 s
5	Hold	4	Indefinitely

BVDV=Bovine viral diarrhea virus

**Figure-1:**
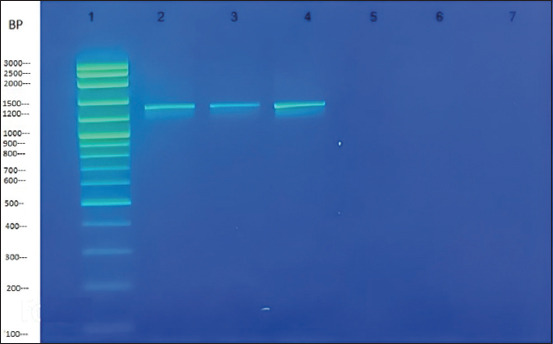
Reverse transcriptase-nested polymerase chain reaction study of the E2 gene for BVDV-1 genetic analysis utilizing blood samples of 1360 bp predicted size according to scale of the marker. (Lane 1: 3000 bp DNA marker; lane 2–4: samples positive for BVDV-1; lane 5: blank; lane 6: non-template control; and lane 7: negative control). BVDV=Bovine viral diarrhea virus.

RNAase-free water served as the negative control. Following gel electrophoresis, the absence of a band in the non-template control confirmed the cleanliness of the master mix. PCR products were displayed on a 1.5% agarose gel stained with SYBR^®^ Safe DNA gel stain (Thermo Fisher Scientific) after being detached using Bio-Rad Gel Documentation Systems. The length of the confirmed BVDV sequences was 1464 base pairs (Figure-1).

### Sanger sequencing and genetic analysis

Using the E2 gene, we sequenced the second-step RT-PCR products, the 3 positive BVDV samples through Sanger method. First BASE Laboratories (FBL) in Malaysia sequenced the selected 3 BVDV double-stranded DNA (dsDNA) PCR products using Sanger sequencing.

In MEGA version 11 (https://www.megasoftware.net/), the BVDV E2 gene sequences were assessed through Sanger sequencing and subjected to genetic analysis. Two new serum isolates from Yogyakarta, Indonesia and 23 reference strains from GenBank were aligned using the MUSCLE technique. 1000 replication bootstraps, Tamura Nei 93 substitution model, and the Gamma G + I distribution, were set for the maximum likelihood method in MEGA 11 for constructing phylogenetic trees. In BioEdit version 7.2.5 (https://bioedit.software.informer.com/7.2/), we evaluated both new isolates’ sequence identities with the reference strains, translated their proteins into amino acids, and identified any point mutations or amino acid changes.

### Statistical analysis

We collected a total of 118 serum samples, among which 10 were positive (Presence of BVDV antigen) and 108 were negative (Absence of BVDV antigen). To determine the proportion of equality between the two groups, we utilized the proportion test on the same set of serum samples (n = 118), which yielded disparate results (positive and negative BVDV samples) for the same population.

### Proportion test

One hundred and eighteen serum samples from each category (positive and negative) were screened using the test findings of the ACE IDEXX. The classification of the total 118 serum samples into first (negative serum samples) and second group (positive serum samples), mainly based on the absence or presence of BVDV antigen, respectively. In the first group, 108 among a total serum samples, unable to show the genetic trait (lack of BVDV antigen), while in the second group, only 10 were BVDV positive among a total of 118 serum samples with a genetic attribute (presence of BVDV antigen).

### Genetic analysis of the E2 glycoprotein

**Figure-2:**
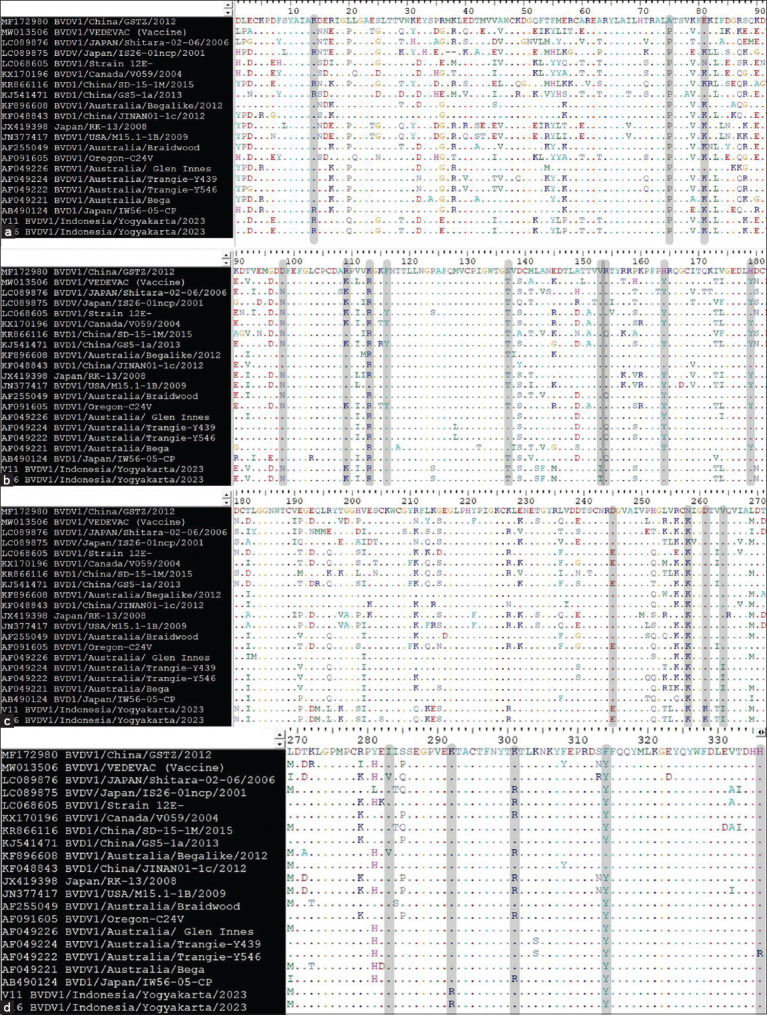
The consensus sequences’ alignment of the recovered two Indonesian isolates (V11 BVDVI/Indonesia/Yogyakarta 2023, and V16 BVDVI/Indonesia Yogyakarta/2023) corresponded with the 19 National Center for Biotechnology Information reference BVDV strains. Amino acid residues were detected in the range (4–295) of the coding region of the BVDV E2 gene. (a–d) The positions of different amino acids of E2 gene in between the range of 1–337 were shaded inside BioEdit software. The two Indonesian isolates have a cysteine mutation to serine (S); this amino acid is still polar at position C201S (c). Multiple alignments of the BVDV E2 gene were generated through Bioedit software [47]. BVDV=Bovine viral diarrhea virus.

It was done descriptively by comparing the data of E2 glycoprotein of the previous researcher with our data by using a statistical program (MEGA). Irianingsih *et al*. [8] found point mutation among conserved cysteine residues inside the E2 glycoprotein, among which the isolate V18-Clp/2015 had a cysteine mutation to glutamine, at number C130G, while the cysteine mutation to serine (S) was found in our research isolates V11 BVDV1/Indonesia/Yogyakarta/2023 and V16 BVDV1/Indonesia/Yogyakarta/2023, this amino acid is still polar at position C201S, as shown in Figure-2. Based on the above facts regarding the occurrence of point mutation in our research findings, the null hypothesis (H_o_) was rejected and the alternative hypothesis (Ha) was accepted (mutation exists). Hence, it is justified that point mutation occurred at different positions of the cysteine residues inside the E2 glycoprotein, leading to the emergence of hypervariability among BVDV isolates, entering into a new animal’s host, vaccination failure, and diagnostic test will not work efficiently.

1000 replication bootstraps and maximum likelihood method were employed with MEGA 11 [46] to construct phylogenetic trees. Twenty three known BVDV strains from National Center for Biotechnology Information (NCBI) GenBank and two BVDV serum samples were aligned using the MUSCLE technique. Using 1000 replication bootstraps and a maximum likelihood technique, phylogenetic trees were created using MEGA 11 [46]. The specific amino acid mutations in the reported and reference BVDV isolates were identified using BioEdit version 7.2.5 based on their alignment and sequence identity [47]. The R language statistical software (R 4.3.3; R Core Team 2021, https://www.r-project.org/) with package ggplot2 was used to conduct the statistical analyses associated with our research [48].

The Datamonkey online tool (www.datamonkey.org) utilized the single-likelihood ancestral counting method to assess non-synonymous and synonymous substitutions in E2 region amino acid sequences.

## Results

### Serological test

**Figure-3:**
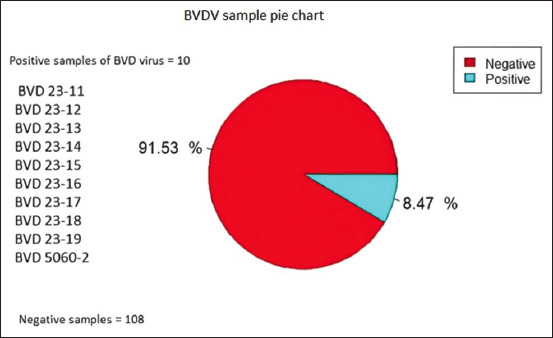
Bovine viral diarrhea serum samples of dairy cattle, negative (108), positive (10).

I-ACE (IDEXX labs Inc., USA) was used to screen 118 sera samples and ten positive BVDV isolates or BVDV-infected serum samples obtained from dairy cattle that had been infected due to BVD virus (Figure-3). All ten positive BVDV serum specimens exhibited OD values ≥0.3 (Table-1).

### Two-step RT-PCR

The two-step RT-PCR result showed that there were ten positive BVDV serum samples. The actual reason that we used a specific pair of primers identical and closely resembling the primer’s sequences of Lang *et al*. [45] and the targeted E2 gene of 3 representative BVDV cDNA isolates was amplified, followed by their band size of 1464 bp after gel electrophoresis (Figure-1, Table-2). The obtained size of the E2 gene was 1464 bp after gel electrophoresis; however, after Sanger sequencing when we inserted forward and reverse sequence files of three BVDV E2 gene isolates inside the alignment editor of MEGA software, we removed the double and non-uniform peaks that create errors in genetic analysis [which possibly occurs due to minor contamination during sequencing] from the BVDV E2 nucleotide length, due to which their size reduced to 1011 bp. We did fine trimming at the location where the graph peak is single and uniform enough between the initial and final points of the alignment editor of the BVDV E2 nucleotides, which is better enough for the ideal phylogenetic analysis.

### BVDV nucleotide sequencing and genetic analysis of the E2 protein

**Figure-4:**
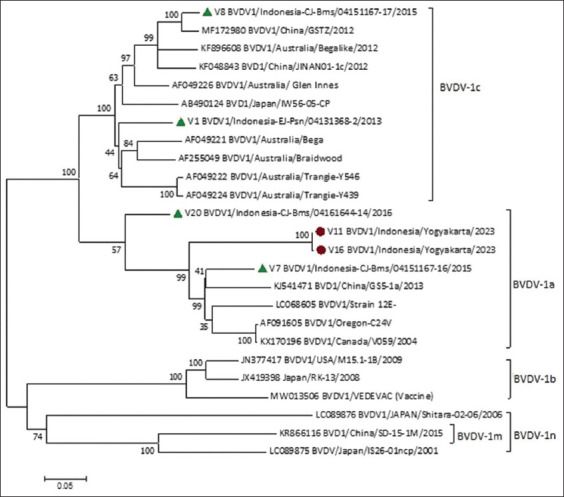
A phylogenetic tree based on the E2 gene (1011 at) that shows the subgenotypes of bovine viral diarrhea virus serum samples recovered from BVDV infected cattle. Using bootstrap values of 1000 replicates, the maximum likelihood approach implemented in molecular evolutionary genetics analysis 11 was used to generate the phylogenetic tree [14].

The characteristic 3 BVDV-positive serum samples with dsDNA aliquots were analyzed using Sanger sequencing at FBL. To determine the subgenotype of the BVDV-1 field samples recovered from Yogyakarta, Indonesia, a phylogenetic tree was constructed using MEGA-11 software, and it was found that the position of the full length of the E2 gene ranged from 2345 to 3356 with a size of 1011 base pair (bp) full nucleotide length of the E2 gene. On the other hand, the position of the E2 gene extended between 2881 and 3218, and its corresponding length was 337 base pairs (bp). Following an E2 gene of the BVDV was targeted during genetic analysis, the phylogenetic tree was constructed from two Indonesian isolates, and 23 reference strains (including 4 Indonesian strains) were downloaded from the NCBI GenBank, which displayed the five subgenotypes BVDV- Ia, BVDV-1b, and BVDV-1c, BVDV-1m, and BVDV-1n (Figure-4). The following 04 BVDV isolates from Indonesia were included in our genetic analysis: V1 BVDV1/Indonesia-EJ-Psn/0413136B-2/2013; V7 BVDV1/Indonesia-CJ-Bms/04151167-16/2015; V8 BVDV1/Indonesia-CJ-Bms/04 151167–17/2015; and V20 BVDV1/Indonesia-CJ-Bms/04 161644–14/2016 [8]. These four Indonesian reference sequences provided a broad overview of our research and were incorporated into the phylogenetic tree. The sequences of our two field strains BVDV-1a (V11 BVDV1/Indonesia/Yogyakarta, Indonesia and V16 BVDV1/Indonesia/Yogyakarta, Indonesia) were inserted in the GenBank and it was found that both sequences of these two isolates 99.90% sequence identity to the BVDV strain Singer (BVDV-1a) (GenBank accession number: AF083348.1). Therefore, based on the report of GenBank as mentioned above, hence, it is justified that peak level (99.90%) of sequence identity exists between our two research BVDV isolates and the BVDV-1a strain Singer-A E2 glycoprotein. However, our third representative BVDV isolate (BVDV 23–13) could not be genetically analyzed due to a 50% missing sequence.

### Sequences of the two BVDV research isolates and their evaluation through GenBank

#### V11 BVDV1/Indonesia/Yogyakarta/2023

The % identity of one of the 2 BVDV sequences V11 BVDV1/Indonesia/Yogyakarta/2023 was calculated with BLAST (http;//blast.ncbi.nlm.nih.gov) (Table-4a).

**Table-4a:** The sequence of one BVDV E2 gene Isolate.

S.No.	Name of BVDV E2 gene Isolate	Sequence of the BVDV E2 gene
1	V11 BVDV1/Indonesia/Yogyakarta/2023	GACTTGGATTGCAAACCTGAATTCTCATATGCCATAGCAAGGGATGA AAGAATTGGTCAACTGGGGGCTGAAGGCCTTACTACCACTTGGAAG GATTACTCGCCTGAAATGAAACTGGAAGACACAATGGTCATAGCTTG GTGCAAAGATGGTAAGTTTACGTACCTCCCAAGGTGCACGAGAGA AACCAGATATCTCGCGATCTTGCATACAAGAGCCTTACCGACCAGTG TGGTATTCAAAAAACTTTTTGATGGGCGAAAGCAAGAGGATGTAGTC GAAATGGACGACAACTTTGAATTTGGACTCTGCCCATGTGA TGCCAAACCCATAGTAAGAGGGAAA TTCAATACAACGCTGCTGAACGGATCGGCCTTCCAGATGGTATGC CCCATAGGATGGACAGGGACTGTAAGCTGTATGTCATTCAATATG GACACCTTAGCCACAACCGTGATACGGACATATAGAAGGTCCAAA CCATTTCCTCATA GGCAAGGCTGTATCACCCAAAAGACTCTGGGGGAGGATCTCCA TAACTGCATCCTCGGAGGAAATTGGACTTGTGTGCCTGGAGACA TGCTATTATACAAAGGGGGCTCTATTGAATCCTGCAAGTGGTGTGG TTATCAATTTAAAGAGAGCGAGGGACTACCACACTACCCCATTGGC AAGTGTAGATTAGAG AATGAGACTGGTTACAGACTAGTAGACGATACCTCTTGTAATAG AGAAGGTGTGGCCATAGTACCACAAGGGACATTACGGTGCAA GATAGGAAAAACTACTATACA GGTCATAGCTATGGATACCAAACTCGGGCCTATGCCTTGCAGAC CATATGAAATAATATCAAGTGAGGGGCCTGTAGAAAGGACAGCG TGTACCTTCAACTACACTAAAACATTAAAAAATAAGTATTTTGAG CCCAGAGACAGCTACTTCC AGCAATACATGCTAAAAGGAGAGTATCAATACTGGTTTGACCTG GAGGTAACCGACCATCACCGG.

BVDV=Bovine viral diarrhea virus

#### V16 BVDV1/Indonesia/Yogyakarta/2023

The % identity of one of the 2 BVDV sequences V16 BVDV1/Indonesia/Yogyakarta/2023 was calculated with BLAST (http;//blast.ncbi.nlm.nih.gov) (Table-4b).

**Table-4b:** The sequence of one BVDV E2 gene Isolate.

S.No.	Name of BVDV E2 gene Isolate	Sequence of the BVDV E2 gene
1	V16 BVDV1/Indonesia/Yogyakarta/2023	GACTTGGATTGCAAACCTGAATTCTCATATGCCATAGCAA GGGATGAAAGAATTGGTCAACTGGGGGCTGAAGGCCTTA CTACCACTTGGAAGGATTACTCGCCTGAAATGAAACTGG AAGACACAATGGTCATAGCTTGGTGCAAAGATGGTAAGT TTACGTACCTCCCAAGGTGCACGAGAGAAACC AGATATCTCGCGATCTTGCATACAAGAGCCTTACCGACCA GTGTGGTATTCAAAAAACTTTTTGATGGGCGAAAGCAAG AGGATGTAGTCGAAATGGACGACAACTTTGAATTTGGAC TCTGCCCATGTGATGCCAAACCCATAGTAAGA GGGAAATTCAATACAACGCTGCTGAACGGATCGGCCTTC CAGATGGTATGCCCCATAGGATGGACAGGGACTGTAAG CTGTATGTCATTCAATATGGACACCTTAGCCACAACCGTG ATACGGACATATAGAAGGTCCAAACCATTTCCT CATAGGCAAGGCTGTATCACCCAAAAGACTCTGGGGGA GGATCTCCATAACTGCATCCTCGGAGGAAATTGGACTTGT GTGCCTGGAGACATGCTATTATACAAAGGGGGCTCTATTG AATCCTGCAAGTGGTGTGGTTATCAATTTAAAG AGAGCGAGGGACTACCACACTACCCCATTGGCAAGTGT AGATTAGAGAATGAGACTGGTTACAGACTAGTAGACGAT ACCTCTTGTAATAGAGAAGGTGTGGCCATAGTACCACAA GGGACATTACGGTGCAAGATAGGAAAAACTAC TATACAGGTCATAGCTATGGATACCAAACTCGGGCCTAT GCCTTGCAGACCATATGAAATAATATCAAGTGAGGGGC CTGTAGAAAGGACAGCGTGTACCTTCAACTACACTAAA ACATTAAAAAATAAGTATTTTGAGCCCAGAGACAGC TACTTCCAGCAATACATGCTAAAAGGAGAGTATCAATAC TGGTTTGACCTGGAGGTAACCGACCATCACCGG.

BVDV=Bovine viral diarrhea virus

### Sequence alignment of the anticipated amino acids

In version 7.2.5 of BioEdit, mutations in specific amino acids were identified, and their sequence identities with reported and reference BVDV isolates were compared. Several alignments depicting the arrangement of the targeted amino acids were also constructed [47]. It was discovered that isolates V11 BVDV1/Indonesia/Yogyakarta/2023 and V16 BVDV1/Indonesia/Yogyakarta/2023 have a cysteine mutation to serine (S); this amino acid is still polar at position C201S (Figure-2c). The positions of different amino acids of E2 gene in between the range of 1–337 were shaded inside BioEdit software (Figures-2a**–**d). This material indicates a shift in the disulfide bond strength characteristic, which is associated with the conserved area of the cysteine amino acid.

**Figure-5:**
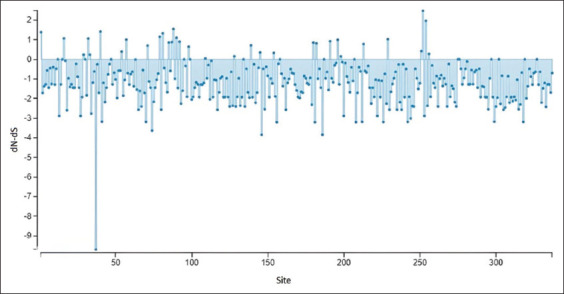
Using the single-likelihood ancestor counting approach, this graph clearly illustrates synonymous and non-synonymous (N) changes in sequences of E2 region amino acids. Variations are prominent at amino acid site 160 till 337, as shown in above figure (www.datamonkey.org).

The results demonstrate that there were differences (Figure-5) encountered in amino acid sequences of the E2 gene region on two sides (between positive and negative graphical zone) and looking predominant between amino acids from site 160 until 337 [49].

## Discussion

A total of 118 serum specimens were collected from dairy cattle affected with BVDV infection and other reproductive disorders followed by screening through ACE (IDEXX) from 2019 to 2023. According to the ACE kit (IDEXX), only ten serum specimens that exhibited an OD value ≥0.3 were considered as positive and confirmed BVDV isolates. However, one BVDV isolate (V13 BVDV1/Indonesia/Yogyakarta/2023) was not genetically analyzed because it lacked the 50% sequence. Still, the two BVDV isolates (V11 BVDV1/Indonesia/Yogyakarta/2023 and V16 BVDV1/Indonesia/Yogyakarta/2023) with accession numbers (GenBank PP836388-PP836389) possessing complete genomic-based information were genetically analyzed by MEGA software to construct the phylogenetic tree. 04 Indonesian reference strains have been added to the phylogenetic tree to provide a more comprehensive overview of the research. The highest bootstrap values, ranging from 99% to 100%, were found for each of the five BVDV subgenotypes with an average pairwise distance of 0.05. The branches of the two BVDV isolates, identified before their separation, share a 99% maximum bootstrap value, indicating a strong confidence level. We observed the branch nearest to our research samples with a bootstrap value of 100, indicating their similarity to our two isolates.

A phylogenetic tree was constructed relying primarily on Sanger sequencing technique’s sequence data during genetic analysis. The positions of the E2 coding fragments (nucleotide positions 2345–3356, 1011 bp) in our study align with Lang *et al*. [45]. Using Sanger’s sequencing data, we constructed a phylogenetic tree with the two Indonesian BVDV1 isolates, four Indonesian BVDV reference strains, and 19 globally distributed BVDV reference strains (all with 100% sequence data found in MEGA software). The resulting phylogenetic tree exhibited the data of 25 BVDV isolates (Indonesian and GenBank reference strains) that were aligned and clustered in BVDV-1 genotype and were subdivided into five subgenotypes of BVDV-1, namely, BVDV-1a, BVDV-1b, BVDV-1c, BVDV-1m, and BVDV-1n. The genetic study also showed that the two BVDV Indonesian isolates were clustered and grouped into the BVDV-1a (n = 2) subgenotype. The Indonesian BVDV subgenotypes 1a, 1b, 1c, 1m, and 1n were found to be homologous with bootstrap values of 100%, 100%, 99%, 100%, and 74%, respectively, and an average pairwise distance of 0.05, after comparison with the BVDV GenBank data. Using MEGA 11 software, the menu of distance was selected, and alignment was inserted to create the distance matrix output between strains of BVDV already mentioned inside the phylogenetic tree. The genetic distance between the pairs of BVDV strains V11 BVDV1/Indonesia/Yogyakarta/2023 and AB490124BVDV1/Japan/W56-05-CP was 0.248, while between V16 BVDV1/Indonesia/Yogyakarta/2023 and AB490124BVDV1/Japan/W56-05-CP was 0.250, the similarity in both pairs of BVDV strains were lower because the value is far from the zero. Similarly, the genetic distance between V11 BVDV1/Indonesia/Yogyakarta/2023 and V16 BVDV1/Indonesia/Yogyakarta/2023 was 0.001; the numerical value is not far from zero; the similarity in this case between our two research isolates was higher. The phylogenetic tree relying on the structural protein E2 with its nucleotide size of 1011 base pairs versus Indonesian BVDV1 isolates and 23 reference sequences from GenBank was displayed (Figure-4). The phylogenetic tree of the E2 region of BVDV1 yielded two subgenotype, BVDV1a and BVDV1c, with a bootstrap value of 99% (Sincere apologies, yes, it was 99%). Two isolates, V11 BVDV1/Indonesia/Yogyakarta/2023 and V16 BVDV1/Indonesia/Yogyakarta/2023, were grouped as subgenotype 1a and entangled with KJ541471 (BVD1/China/GS5-1a/2013). The BVDV E2 sequences generated using MEGA 11 software revealed that 167/337 (49.55%) and 579/1011 (57.27%) of the sides varied at the amino acid and nucleotide levels, respectively. Eighty-seven single variable sites, 492 Parsimony informative sites, and 432 conserved sites were noted at the nucleotide level.

About 49% or more of nucleotides were found at positions 10–248 and 278–1006. The conversed regions were primarily located at amino acid positions 60–81, 96–139, and 258–314, despite numerous changes at other positions. Our sequences had a greater rate of amino acid substitution than the prevailing CSFV in China [50], implying they may have evolved more rapidly.

Because E2 is the primary immunogen of the BVDV, it is the most variable protein in the ORF. Based on research by Ridpath [51] and Neill [52], the N-terminus of the hypervariable region of the E2 protein contains a neutralizing epitope. Vaccination failure is partly caused by antigenic differences in the E2 protein across BVD strains [51]. The two characteristic isolates of our research are V1 V11 BVDV1/Indonesia/Yogyakarta/2023 and V16 BVDV1/Indonesia/Yogyakarta/2023, as they belong to the BVDV-1a subgenotype, based on the E2 region. This permits the formation of modifications to the E2 (gp53) protein. The E2 protein of BVDV is significant for the development of vaccines and medical equipment for diagnosis. Deregt and Prins [53] and Li *et al*. [54] reported that the E2 gene has 17 invariant cysteine residues located at positions 4, 48, 59, 104, 106, 130, 140, 168, 181, 189, 201, 208, 226, 242, 257, 278, and 295. The isolates V11 BVDV1/Indonesia/Yogyakarta/2023 and V16 BVDV1/Indonesia/Yogyakarta/2023 were identified to have a cysteine mutation to Serine (S); this amino acid remains polar at position C201S, as shown in Figure-2. Within the E2 glycoprotein, point mutations were discovered by Irianingsih *et al*. [8]; of them, isolate V18-Clp/2015 exhibited a cysteine mutation to glutamine at position C130G. It follows that point mutations at various locations of the cysteine residues within the E2 glycoprotein are justified, as this causes the establishment of hypervariability among BVDV isolates, entry into a new animal host, failure of vaccination, and ineffectiveness of diagnostic tests.

This material indicates a change in the disulfide bond strength characteristic, which is connected to the cysteine amino acid’s conserved area [53, 54].

**Figure-6:**
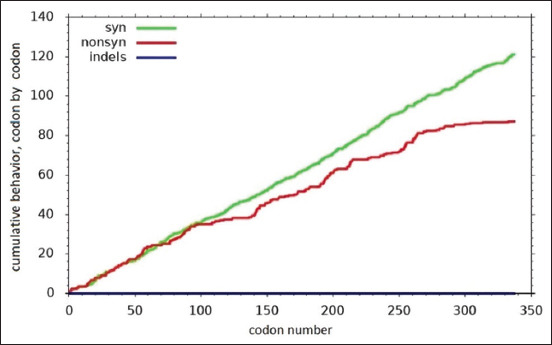
Synonymous (dS) alterations are more abundant than non-synonymous (N) changes (23).

The dN/dS ratio of the E2 area yields a negative value (p < 3.6961) for each of these sides (Table-5). Analysis of the E2 BVDV gene alignments revealed quite noticeable variants on the E2 gene (Table-5). It displays a negative result graphically as a Dn/dS value <0 (dN/dS) because synonymous substitutions representing the evolutionary replacement substitution of one base for another in an axon coding of a gene expression into protein are nonetheless more common than non-synonymous substitutions (Figure-6) [55]. This suggests that the E2 gene is highly variable (hypervariable), with a reported value of dN/dS comparison threshold of 0.207 as determined by other researchers [56]. Glycosylated viral proteins significantly influence the pathogenicity and multiplication of viruses in their natural hosts. 7D, 9S, 32D, and 70TRALPTS76 are conserved residues that contain antigenic sites [45]. According to our research, isolates V11 BVDV1/Indonesia/Yogyakarta/2023 and V16 BVDV1/Indonesia/Yogyakarta/2023 exhibited alterations in the D7E residues.

**Table-5:** The E2 area’s dN/dS ratio yields negative values of 0.0047 and 0.0013, indicating a higher prevalence of synonymous (dS) alterations compared to non-synonymous (dN) changes.

Compare	Sequence names	Sd	Sn	S	n	ps	Pn	ds	dn	ds/dn	Ps/pn
1	*V11_BVDV1/Indonesia/Yogyakarta2013 *V16_BVDV1/Indonesia/Yogyakarta2013	0.000	1.000	215.666	795.333	<0.0046	0.0013	<0.0047	0.0013	<3.696	<3.687

The E2 protein functions as a glycoprotein connected to the envelope, immunodominant structural proteins, and membrane-bound proteins. Its protein structure comprises of 4–6 glycosylation sites [51]. Because there are so many different types of tall regions, Gene E2 is a less sustainable region. This study’s analysis of the alignment of two new isolates and 23 GenBank references revealed 190/337 variations in amino acid levels. Residues 4–87 of the DA domain, 88–164 of the DB domain, 165–271 of the DC domain, and 272–333 of the DD domain are the four domains that make up the E2 protein. These domains are ordered linearly from the N–to the C–terminus [25]. Li *et al*. [54] reported that the E2 protein has three domains: Domain I, which consists of residues 693–782, domain II, which includes residues 783–860, and domain III, which comprises residues 861–1036. These domains have slightly distinct functions. When attached to host cell receptors, the immunoglobulin-like fold of the DA and DB domains is constant. The pH circumstances (6–6.4) impact the conserved H70 residue in the DA domain [54]. The consecutive glycosylation positions in the DB, DC, and DD motifs are N117, N186, and N230 and N286. According to El Omari *et al*. [57], homodimerization of the DD region with cysteine residues is crucial for host cell invasion. Analysis of the two isolates of the BVDV revealed no alterations in the cysteine residues.

## Conclusion

Based on genetic analysis of their E2 gene, the recovered BVDV isolates were clustered into BVDV-1a subgenotype inside the phylogenetic tree. Our investigation indicates that the D7E conserved residues were altered in both V11 BVDV1/Indonesia/Yogyakarta/2023 and V16 BVDV1/Indonesia/Yogyakarta/2023 isolates. In isolates from Indonesia, a mutation from cysteine (C) to serine (S) at position 201 in the BVDV sequence was identified, which consequently resulted in failure of vaccine, incapability of diagnostic test for BVDV screening, and range of animal’s host will increase. Therefore, hence it is verified based on our above facts and findings, it is highly recommended that researchers across the worldwide need to strengthen their efforts to conduct research regarding the occurrence of mutation inside E2 hypervariable region of BVDV to formulate an effective vaccine for eradication of BVD virus globally.

## Authors’ Contributions

SUK: Conducted, conceptualized, planned, designed, and evaluated the study. HW, MHW, SS, and SHI: Supervised the study and drafted and revised the manuscript. All authors have read, reviewed, and approved the final manuscript.
